# The Value of Multimodal Ultrasound in Differentiating Benign from Malignant Cytologically Indeterminate Thyroid Nodules

**DOI:** 10.3390/cancers18071071

**Published:** 2026-03-25

**Authors:** Rong Yang, Yanfang Wang, Guo Chen, Xiaorong Lv, Yuanqing Zhang, Fang Nie

**Affiliations:** 1 Ultrasound Medical Center, The Second Hospital of Lanzhou University, Cuiyingmen No. 82, Chengguan District, Lanzhou 730030, China; yangr2023@lzu.edu.cn (R.Y.);; 2 Gansu Province Clinical Research Center for Ultrasonography, Lanzhou 730030, China; 3 Gansu Province Medical Engineering Research Center for Intelligence Ultrasound, Lanzhou 730030, China; 4 Gansu Province Interventional Ultrasound Equipment Application Industry Technology Center, Lanzhou 730030, China

**Keywords:** thyroid cancer, indeterminate thyroid nodules (ITNs), Bethesda III, contrast-enhanced ultrasound, conventional ultrasound, FNAB, risk stratification

## Abstract

Aim: To evaluate the diagnostic value of conventional ultrasound (CUS) and contrast-enhanced ultrasound (CEUS) features in differentiating benign from malignant Bethesda III/IV thyroid nodules, and to identify independent predictors of malignancy. Methods: We retrospectively analyzed 164 surgically confirmed Bethesda III/IV thyroid nodules. CUS and CEUS features were evaluated by two experienced radiologists blinded to pathological outcomes. Univariate analysis compared features between benign and malignant groups. Multivariate logistic regression was used to identify independent predictors. Diagnostic models were constructed based on CUS alone, CEUS alone, and their combination, with performance evaluated using receiver operating characteristic (ROC) curve analysis. The area under the curve (AUC), sensitivity, specificity, positive predictive value (PPV), and negative predictive value (NPV) were calculated for each model. Results: The malignancy rate was 48.8% (80/164). Multivariate analysis identified microcalcifications (OR = 4.815, *p* < 0.001), aspect ratio >1 (OR = 2.499, *p* = 0.028), and irregular shape (OR = 2.465, *p* = 0.035) as independent risk factors, while older age (OR = 0.926 per year, *p* < 0.001) was protective. The CUS model achieved an AUC of 0.815 with high sensitivity (91.3%) and NPV (87.7%). The CEUS model performed poorly (AUC = 0.609). The combined model (AUC = 0.823) showed no significant improvement over CUS alone (*p* > 0.05). Physician subjective diagnosis based on CEUS TI-RADS yielded an AUC of 0.775. Conclusions: Conventional ultrasound features provide good diagnostic value for Bethesda III/IV nodules, with high sensitivity and NPV suitable for clinical screening. The addition of CEUS offered limited incremental benefit in this specific population, suggesting that the diagnostic value of CEUS for differentiating benign from malignant cytologically indeterminate thyroid nodules (ITNs) may be limited.

## Simple Summary

Thyroid nodules are commonly found during routine physical examinations, but most are benign. In about 20% of cases where a patient undergoes a thyroid biopsy, the results still cannot clearly determine whether a nodule is benign or malignant (classified as Bethesda III/IV), posing a dilemma for doctors and patients: should they opt for surgical removal or continued observation? This study evaluated the value of two ultrasound techniques—conventional ultrasound (CUS) and contrast-enhanced ultrasound (CEUS)—in addressing this challenge. We retrospectively analyzed 164 surgically confirmed nodules of this type and found that certain features on conventional ultrasound (such as microcalcifications and irregular shape) effectively predict malignancy risk with high screening sensitivity. However, contrast-enhanced ultrasound did not significantly improve diagnostic accuracy. The public health significance of this finding is that for patients with inconclusive biopsy results, conventional ultrasound provides more reliable reference information than contrast-enhanced ultrasound, helping doctors and patients collaboratively make more precise decisions, avoid unnecessary surgeries, and achieve personalized management of thyroid nodules.

## 1. Introduction

The widespread application of conventional ultrasound (CUS) has led to a gradual increase in the detection rate of thyroid nodules (TNs). However, only approximately 7–15% of these nodules are malignant [1]. Consequently, accurately distinguishing between benign and malignant thyroid nodules has become a primary focus in the clinical diagnosis and management of TNs. Owing to its non-invasive nature, convenience, and cost-effectiveness, CUS has emerged as the preferred initial imaging modality for thyroid evaluation in clinical practice. To further enhance diagnostic accuracy, various institutions have proposed different versions of the Thyroid Imaging Reporting and Data System (TI-RADS) [2,3]. Nevertheless, certain benign and malignant nodules cannot be differentiated by CUS. For instance, both solid composition and hypoechogenicity can be sonographic features common to both benign and malignant nodules.

Ultrasound-guided fine-needle aspiration biopsy (FNAB) is currently one of the most accurate methods for evaluating the nature of thyroid nodules and is recommended by the American Thyroid Association [4]. According to the 2023 Bethesda System for Reporting Thyroid Cytopathology (TBSRTC) [5], Bethesda category III and IV nodules are classified as Cytologically Indeterminate Thyroid Nodules (ITNs). Their diagnosis falls between benign and malignant, precluding a definitive diagnosis [6]. Studies indicate that such indeterminate nodules account for 15–25% of all FNAB results [7]. Even repeat FNAB fails to yield a definitive diagnosis in approximately 20% of cases [8]. Furthermore, the interpretation of Bethesda III cytology itself is subject to significant interobserver variability. A recent study using the 2023 Bethesda System for Reporting Thyroid Cytopathology (TBSRTC) reported only moderate interobserver agreement for Bethesda III nodules (κ = 0.429), substantially lower than the good agreement observed for benign category (κ = 0.606) [9]. This inherent subjectivity further complicates clinical decision-making and underscores the need for complementary objective diagnostic tools. Therefore, it is necessary to explore alternative approaches to address this diagnostic dilemma.

Contrast-Enhanced Ultrasound (CEUS) is an imaging technique capable of dynamically observing tissue microcirculation perfusion. In the differentiation of benign and malignant thyroid nodules, CEUS can more effectively depict the intranodular blood flow distribution and perfusion patterns compared to CUS, potentially offering an advantage [10]. However, the current high-quality evidence regarding the use of CEUS for determining the benign or malignant nature of cytologically indeterminate thyroid nodules (Bethesda category III/IV thyroid nodules) remains very limited. There is even less domestic research on this topic, and no unified conclusion has been reached. Therefore, this study aims to systematically analyze the diagnostic value of CUS and CEUS features in differentiating benign from malignant Bethesda category III/IV thyroid nodules, and to screen for independent predictors of malignant risk. The goal is to provide a useful reference for the clinical stratified management and decision-making regarding Bethesda category III/IV thyroid nodules.

## 2. Materials and Methods

**Figure 1:**
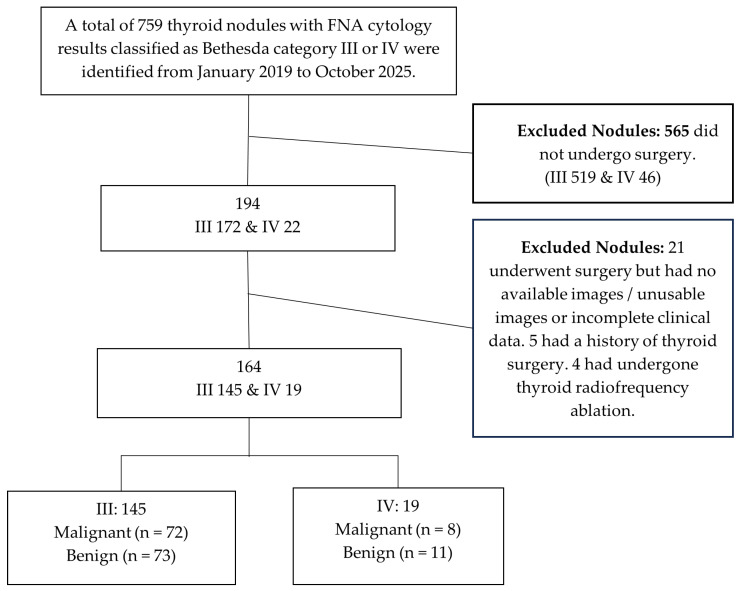
Patient Selection Flowchart.

This retrospective study was approved by our Institutional Review Board (Project Number: 2024A-530), and the informed consent was obtained from all subjects involved in the study. All study procedures adhered to the ethical principles of the Declaration of Helsinki. Consecutive cases with thyroid nodules diagnosed as Bethesda category III or IV by FNAB and with subsequent surgical pathological results at our hospital from January 2019 to October 2025 were enrolled. Inclusion criteria were: (1) Having undergone both CUS and CEUS examinations; (2) Having undergone FNA with a cytopathological result of Bethesda category III or IV; (3) Possessing qualified ultrasound image quality and complete clinical data; (4) No history of thyroid surgery, ablation therapy, or radioiodine examination prior to FNA; (5) Age ≥18 years. Exclusion criteria were: (1) Incomplete preoperative thyroid ultrasound and clinical data, or poor image quality; (2) Unclear final postoperative pathological diagnosis; (3) Allergy to the SonoVue (Bracco Imaging S.p.A., Milan, Italy) microbubble contrast agent; (4) Pregnancy; (5) Severe cardiac, hepatic, or renal dysfunction. Patient demographics, ultrasound images, cytological, and histopathological reports were extracted from our hospital’s electronic medical record system, Picture Archiving and Communication System (PACS), and Hospital Information System (HIS). The case selection flowchart is detailed in Figure 1.

All ultrasound examinations were performed using a Siemens ACUSON Sequoia system (Siemens Healthineers, Erlangen, Germany) with an L12-3 probe (frequency 4–10 MHz) and a Canon Aplio i800 system (Canon Medical Systems Corporation, Otawara, Japan) with an i18LX5 probe (frequency 5–18 MHz). Patients were positioned supine with the neck fully exposed. Examinations were conducted by experienced sonographers with over 10 years of experience in thyroid intervention. For each nodule, static and dynamic images in both longitudinal and transverse planes were stored. CUS features including size, composition, echogenicity, aspect ratio, margin, and calcifications were recorded. CEUS was performed using the second-generation microbubble contrast agent SonoVue, whose main component is sulfur hexafluoride-filled microbubbles. Each patient underwent CEUS examination using the same equipment mentioned above. The patient’s position remained unchanged, and the same target thyroid nodule was selected. After the nodule’s largest cross-section was displayed, the ultrasound system was switched to CEUS mode. A rapid bolus injection of 2.4 mL contrast agent followed by a 5 mL saline flush was administered via the antecubital vein. The scanning plane was kept fixed, and patients were instructed to avoid swallowing or moving. Continuous observation and recording of dynamic images were performed for 150 s. Both CUS and CEUS data were stored on the local hard drive and immediately transferred to a dedicated external hard drive for subsequent analysis. Both CUS and CEUS images were independently evaluated by two experienced radiologists who were blinded to subsequent follow-up, cytological, or surgical histopathological diagnoses. Both radiologists had over 10 years of experience in thyroid imaging. In cases where discrepancies arose, consensus was reached through discussion and joint re-evaluation of the images, and this consensus interpretation was used for the final analysis. This approach is widely accepted in similar retrospective imaging studies, as it helps minimize individual bias while reflecting real-world clinical practice where difficult cases are often reviewed collaboratively.

All FNA procedures were performed by the same sonographer following the CEUS examination. Patients were positioned supine, breathing calmly, with the neck fully exposed. The skin over the anterior neck was routinely disinfected and draped. Under real-time ultrasound guidance, a 25 G*50 mm needle (Becton, Dickinson and Company, Franklin Lakes, NJ, USA) was used. The needle path was selected to avoid adjacent critical vessels and organs. After the needle entered the nodule, a syringe with negative pressure was connected. Aspiration was performed with 5–10 back-and-forth movements within different parts of the nodule before rapid needle withdrawal. The aspirated material was smeared and fixed in 95% alcohol for interpretation by cytopathologists. In cases of discordant cytological results, the final diagnosis was made after review by the chief cytopathologist. All cases were reported using the 2023 Bethesda System for Reporting Thyroid Cytopathology (TBSRTC) 5. Postoperative pathological examination served as the gold standard. All cytology slides were reviewed by two experienced cytopathologists at our institution, and consensus was reached for cases with initial disagreement, which helps minimize but cannot eliminate this inherent subjectivity.

First, two radiologists assessed the conventional ultrasound features of each nodule according to C-TIRADS criteria and assigned an initial TI-RADS category. Subsequently, the same radiologists analyzed the CEUS dynamic images, focusing on enhancement patterns, homogeneity, and margins. The integrated diagnosis was performed following these rules: If CEUS showed malignant features such as heterogeneous hypo-enhancement or ill-defined enhancement margins, the initial TI-RADS category was upgraded by one level. If CEUS showed homogeneous iso-/hyper-enhancement, the initial category was downgraded by one level. If there was no clear tendency, the initial category remained unchanged. The final integrated TI-RADS category (with ≥4b considered malignant) was used for subsequent analysis.

All statistical analyses were performed using IBM SPSS Statistics software (Version 29.0; IBM Corp). Measurement data are presented as mean ± standard deviation, and count data as number (percentage). The normality of continuous variables was assessed using the Shapiro–Wilk test. Based on data type and distribution characteristics, intergroup comparisons were performed using the following methods: independent samples t-test for normally distributed continuous variables, Mann–Whitney U test for non-normally distributed continuous variables; Chi-square test or Fisher’s exact test for categorical variables. Multivariate binary logistic regression analysis was employed to identify independent predictors associated with malignant risk, calculating odds ratios and 95% confidence intervals. Receiver operating characteristic (ROC) curves were plotted to evaluate the discriminative efficacy of different diagnostic models. The area under the curve (AUC), sensitivity, and specificity were calculated. The Z-test was used to compare differences between AUCs. Using the CEUS-TIRADS classification as a standard, a category of ≥4b was defined as malignant and compared with the US-CEUS integrated model developed in this study. The diagnostic accuracy of subjective assessment by radiologists was also compared with that of the model. A *p*-value < 0.05 was considered statistically significant.

## 3. Results

### 3.1. Baseline Characteristics

A total of 164 surgically confirmed Bethesda category III and IV thyroid nodules were ultimately included in this study. Patient baseline data are presented in Table 1. There were 80 malignant nodules (48.8%) and 84 benign nodules (51.2%). The pathological results are shown in Table 2. Patients in the malignant group were significantly younger than those in the benign group (44.5 ± 10.8 years vs. 51.7 ± 9.3 years, *p* < 0.001), and their nodules had a smaller maximum diameter (8.4 ± 7.2 mm vs. 13.3 ± 10.8 mm, *p* < 0.001). There was also a difference in gender distribution between the two groups, with a higher proportion of females in the malignant group (92.5% vs. 78.6%, *p* = 0.012). There were no significant differences in TSH or Tg levels between the groups (*p* > 0.05).

### 3.2. Univariate Analysis

**Figure 2:**
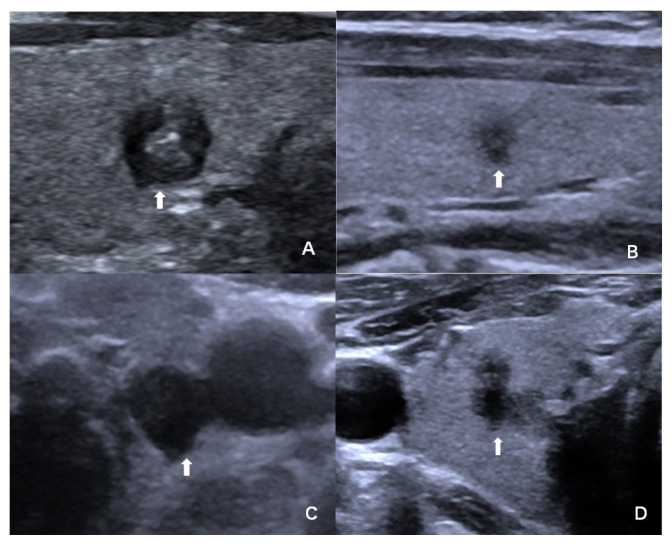
The white arrow indicates the thyroid nodule. (**A**). Female, 46 y, Papillary Thyroid Carcinoma (PTC), Bethesda Category III. A solid hypoechoic nodule measuring 5 mm × 5 mm × 6 mm in the right thyroid lobe with a taller-than-wide shape (aspect ratio >1) and internal microcalcifications. (**B**). Female, 42 y, Bethesda Category III, PTC. A solid hypoechoic nodule measuring 3 mm × 2 mm × 4 mm in the right thyroid lobe with a taller-than-wide shape and irregular margin. (**C**). Female, 60 y, Bethesda Category IV, Papillary Thyroid Carcinoma. A solid hypoechoic nodule measuring 8 mm × 6 mm × 6 mm in the left thyroid lobe with a taller-than-wide shape and irregular margin. (**D**). Male, 35 y, Bethesda Category III, PTC. A solid hypoechoic nodule measuring 4 mm × 3 mm × 6 mm in the right thyroid lobe with a taller-than-wide shape and irregular shape.

**Figure 3:**
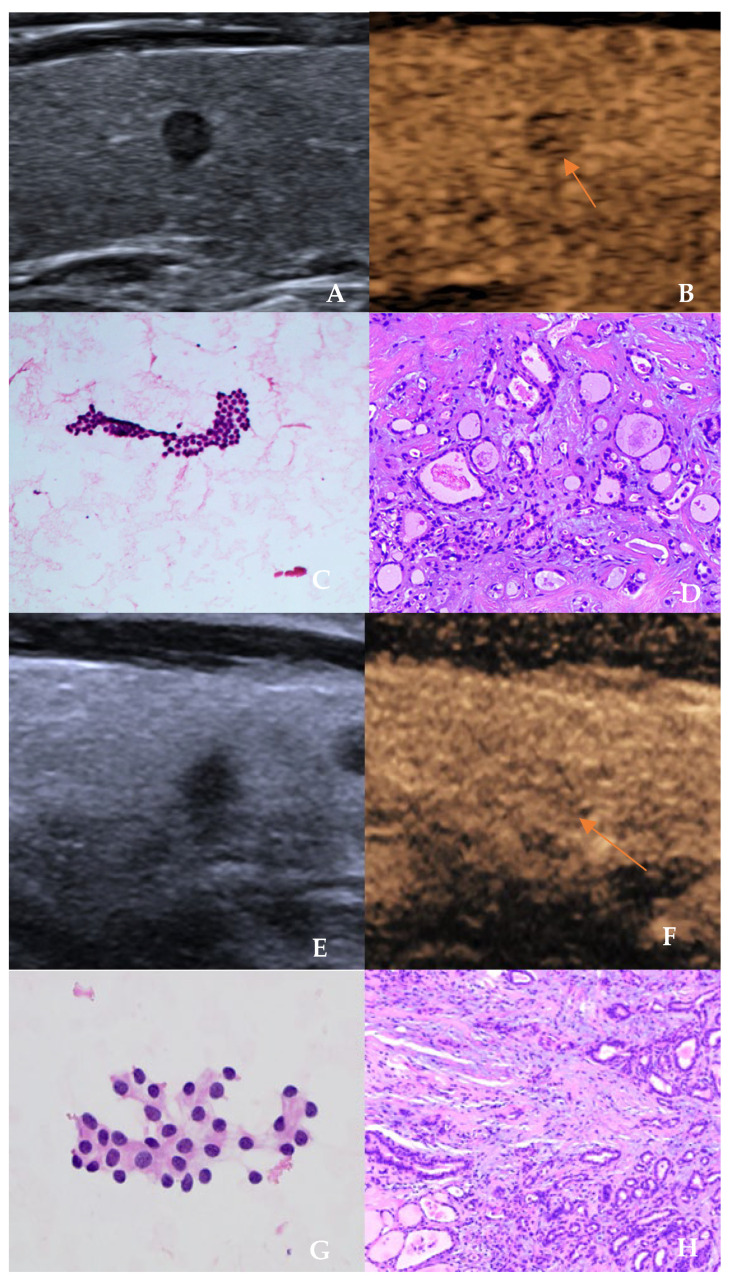
Ultrasound and pathological images of two patients with papillary thyroid carcinoma. (**A**–**D**): A 58-year-old female patient. (**A**): Two-dimensional ultrasound shows a solid hypoechoic nodule in the middle of the right lobe (3 mm × 3 mm × 4 mm), with a taller-than-wide shape (aspect ratio > 1), classified as C-TIRADS 4c; (**B**): Contrast-enhanced ultrasound shows low enhancement in the wash-out phase (orange arrow); (**C**): Fine-needle aspiration cytology (Bethesda category III, HE ×200); (**D**): Postoperative histopathology confirms papillary thyroid carcinoma (HE ×200). (**E**–**H**): A 60-year-old male patient. (**E**): Two-dimensional ultrasound shows a solid hypoechoic nodule in the right lobe (4 mm × 4 mm × 3 mm); (**F**): Contrast-enhanced ultrasound shows iso-enhancement in the wash-out phase (orange arrow); (**G**): Fine-needle aspiration cytology (HE ×200); (**H**): Postoperative histopathology confirms papillary thyroid carcinoma (HE ×200).

The comparison of ultrasound features between benign and malignant nodules is detailed in Table 2. Regarding conventional ultrasound features, malignant nodules were more frequently associated with solid composition, hypoechogenicity, taller-than-wide shape (aspect ratio ≥1), ill-defined margin, presence of microcalcifications, irregular shape (as shown in Figure 2), and absence of a halo (all *p* < 0.05). Among CEUS features, only “ill-defined enhancement margin” (as shown in Figure 3) was significantly associated with malignancy (81.3% vs. 59.5%, *p* = 0.002). Other CEUS parameters (e.g., direction of enhancement, degree of washout, enhancement homogeneity) showed no significant differences between the two groups (*p* > 0.05). Furthermore, ROC curve analysis determined the optimal cut-off values for age and nodule maximum diameter in differentiating benign from malignant nodules. Using the maximum Youden index as the criterion, the optimal cut-off for age was ≤50.5 years, with a diagnostic sensitivity of 70.0%, specificity of 66.7%, and accuracy of 68.3%. The optimal cut-off for nodule maximum diameter was ≤7.5 mm, with a diagnostic sensitivity of 63.8%, specificity of 70.2%, and accuracy of 67.1%. The positive and negative predictive values for both parameters applied individually ranged between 67% and 70%.

### 3.3. Multivariate Analysis Results

Variables with *p* < 0.05 in the univariate analysis were included in a binary logistic regression model, with results shown in Table 3. After controlling for other variables, age, microcalcifications, aspect ratio > 1, and irregular shape were identified as independent factors for differentiating benign from malignant ITNs. Among these, age was negatively correlated with malignant risk (OR = 0.926, 95% CI: 0.888–0.965, *p* < 0.001), meaning the risk of malignancy decreased by approximately 7.4% on average for each one-year increase in age. Microcalcifications were the strongest independent imaging marker for predicting malignancy; nodules with microcalcifications had a 4.815-fold higher malignant risk compared to those without calcifications (95% CI: 1.953–11.871, *p* < 0.001). Additionally, aspect ratio >1 (OR = 2.499, 95% CI: 1.101–5.672, *p* = 0.028) and irregular shape (OR = 2.465, 95% CI: 1.067–5.694, *p* = 0.035) also independently indicated a higher risk of malignancy. However, ill-defined enhancement margin, gender, nodule maximum diameter, ill-defined margin alone, and macrocalcifications did not demonstrate a statistically independent association with nodule malignancy risk in this multivariate model.

### 3.4. Diagnostic Performance of Each Model

**Figure 4:**
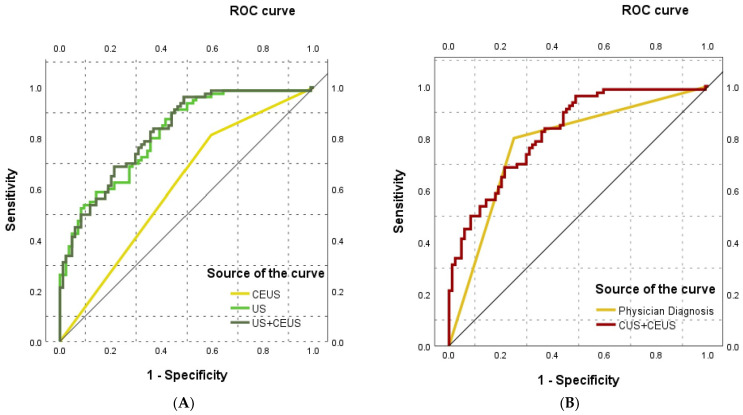
ROC curves. (**A**) ROC curves of the three ultrasound-based diagnostic models. (**B**) ROC curves comparing the physician’s diagnosis and the combined model.

This study systematically evaluated the diagnostic efficacy of the conventional ultrasound model, the CEUS model, the combined model, and the subjective diagnosis by radiologists (based on CEUS TI-RADS criteria) for differentiating ITNs (as shown in Figure 4). The combined model demonstrated the highest overall diagnostic performance, with an area under the curve (AUC) of 0.823 (95% CI: 0.761–0.885), slightly higher than the AUC of 0.815 (95% CI: 0.752–0.879) for the conventional ultrasound model. The subjective diagnosis by radiologists yielded an AUC of 0.775 (95% CI: 0.701–0.849), while the standalone CEUS model had the lowest diagnostic efficacy (AUC = 0.609, 95% CI: 0.522–0.695) (as shown in Table 4).

## 4. Discussion

It is currently established that FNAB is the gold standard for preoperative detection and exclusion of cancer [11] and is recommended by multiple guidelines both in China and internationally [12]. FNAB can effectively reduce unnecessary surgeries, improve patient prognosis, and significantly enhance the accuracy of preoperative evaluation for thyroid nodules. However, FNAB results alone cannot definitively determine the benign or malignant nature of cytologically indeterminate thyroid nodules. Comprehensive judgment typically requires integration with ultrasound features, molecular testing, and follow-up outcomes. Ultrasound, as one of the auxiliary diagnostic tools for FNAB, holds certain diagnostic value in thyroid nodule assessment. Specifically, two-dimensional ultrasound can preliminarily screen for malignancy risk based on nodule morphology, margin, and calcifications, but its diagnostic efficacy is influenced by observer subjectivity and has limited discriminative power for atypical nodules. Conversely, CEUS reflects nodule vascular characteristics from the perspective of dynamically observing microvascular perfusion. Nonetheless, regarding the specific population of ITNs, existing studies predominantly focus on the application of a single technique [13,14]. Therefore, this study primarily aims to systematically evaluate the consistency between CUS, CEUS features, and pathological results, thereby exploring their clinical value in differentiating benign from malignant indeterminate thyroid nodules.

CEUS is an advanced technique developed in recent years, offering advantages such as being non-invasive, radiation-free, and non-nephrotoxic. It can detect blood flow within vessels as small as <100 μm in diameter, allowing real-time dynamic observation of thyroid nodule perfusion, making it a commonly used imaging method for thyroid nodules [15]. This study comprehensively compared and analyzed the diagnostic value of CUS, CEUS, and their combined model in differentiating benign from malignant ITNs. The results indicated that the CUS model demonstrated the best “rule-out” capability (AUC = 0.815, sensitivity 91.3%), making it suitable for high-sensitivity clinical screening. The CEUS model performed the worst (AUC = 0.609), and the combined model (AUC = 0.823) showed only a 1.8% numerical improvement over the CUS model. However, its sensitivity (83.8%) was lower than that of the CUS model, and its specificity (61.9%) did not surpass the subjective diagnosis by radiologists based on CEUS TI-RADS (AUC = 0.775, specificity 75.0%). This suggests that physicians’ experience plays a crucial role in integrating complex imaging information and making accurate “confirmatory” decisions. Their diagnostic specificity reached 75.0%, indicating a good ability to identify benign lesions and potentially prevent unnecessary interventions. In contrast to findings from some studies [6,16,17] suggesting that CEUS significantly improves diagnostic specificity, the present study indicates that CEUS offers limited diagnostic value for cytologically indeterminate thyroid nodules. This may be attributed to the inherent subjectivity of qualitative CEUS parameter interpretation, which could have reduced its diagnostic performance. Furthermore, the considerable pathological heterogeneity of Bethesda III/IV nodules means that CEUS performance may vary across different histological subtypes, potentially diluting its overall discriminative ability in this diverse population. Although univariate analysis revealed a significant association between “ill-defined enhancement margin” and malignancy, this feature was not an independent predictor in multivariate analysis. This may be because the microvascular perfusion information provided by CEUS (e.g., “ill-defined enhancement margin”) substantially overlaps with the high-resolution morphological features of CUS (e.g., “ill-defined margin”). Additionally, ITNs inherently possess considerable pathological heterogeneity, and the interpretation of CEUS involves a degree of subjectivity, which may also have impacted its discriminative efficacy. This challenge is compounded by the moderate interobserver reproducibility of the Bethesda III cytology itself (κ = 0.429) [9]. The heterogeneity inherent in cytologically indeterminate nodules—both in their pathological subtypes and in the cytological interpretation—may dilute the apparent diagnostic performance of any adjunctive imaging modality, including CEUS. Simultaneously, a recent study by Gokozan et al. [18] demonstrated that interobserver agreement for Bethesda III varies significantly and is influenced by reviewer experience, with more experienced cytopathologists showing higher concordance rates. In addition, the study by Liu et al. [6] included a high proportion of Bethesda V nodules (63.8%), which often exhibit more pronounced malignant features on CUS. In such cases, CEUS may provide additional microvascular perfusion information, potentially leading to higher diagnostic performance (AUC = 0.848). Our cohort consisted solely of ITNs. Given their relatively lower malignancy risk and less conspicuous conventional ultrasound features, the perfusion information contributed by CEUS was also limited, resulting in a more modest diagnostic role.

Given that the model incorporates established malignant risk factors supported by extensive multivariate analyses—including microcalcifications, taller-than-wide shape (aspect ratio >1), and irregular shape—all three of which are independent predictors of nodule malignancy. While some studies suggest that ultrasound features cannot reliably predict the malignancy of indeterminate thyroid nodules [19], our findings align with the majority of published literature [20,21,22,23]. When any one of these three indicators is present in a nodule, the likelihood of malignancy significantly increases. Furthermore, within our cohort of patients already meeting surgical indication criteria (presence of suspicious ultrasound features or strong patient preference), we observed a malignancy rate of 48.8% (80/164) for ITNs. This indicates that even within this high-risk subgroup, more than half of the nodules were ultimately benign, suggesting that proceeding directly to surgery would lead to overtreatment. Contrary to the view of Batawil et al. [24] that “surgery should be the first choice for indeterminate nodules,” we advocate for more refined risk stratification in managing such nodules. This includes integrating molecular testing for better stratification, recommending surgery for high-risk cases, and implementing active surveillance for low-risk, indolent nodules. This approach is beneficial for minimizing unnecessary surgical complications (such as hypoparathyroidism) and may offer potential advantages, such as thermal ablation techniques (e.g., microwave ablation), for suitable candidates, thereby maximizing overall patient benefit.

Concurrently, the study identified age as an independent protective factor against thyroid nodule malignancy (OR = 0.926, *p* < 0.001), aligning with the epidemiological characteristics of papillary thyroid carcinoma (predilection for younger patients) [23,25]. This suggests that patient age serves as a risk-calibrating factor in the evaluation of ITNs. For cytologically indeterminate nodules in younger patients (<50.5 years), even with atypical ultrasound appearances, the inherent age-related risk confers a relatively higher malignancy probability, warranting more careful consideration regarding the need for more aggressive diagnostic or follow-up strategies. Conversely, in older patients, nodules are more likely to exhibit lower biological activity; in such cases, extending follow-up intervals or reducing frequency may be appropriate.

Secondly, although nodule size was not an independent predictor of malignant risk, its highly significant *p*-value (<0.001) in univariate analysis indicates a substantial association with benign/malignant differentiation. Consistent with reports such as that by Zhang et al. [26], malignant nodules in our study tended to be smaller (optimal cutoff ≤7.5 mm). Most versions of the Thyroid Imaging Reporting and Data System (TI-RADS) incorporate nodule size in biopsy decision-making. Existing evidence suggests that nodule size influences the diagnostic accuracy of FNAB [27,28]: for nodules <1 cm in diameter, factors such as limited cellular yield, higher non-diagnostic rates, and increased cytological false-negative rates may compromise FNAB performance. Therefore, for ITNs with elevated malignancy risk in our study, “small size” might be an intrinsic characteristic, potentially contributing to the limited accuracy of FNAB and resulting in an “indeterminate” diagnosis. Over-reliance on cytology for small nodules, without fully considering high-risk morphological features on conventional ultrasound (e.g., microcalcifications, taller-than-wide shape), can complicate clinical decision-making. This implies that small nodules (≤7.5 mm) lacking high-risk morphological features may be more indolent. However, vigilance is warranted when small nodules exhibit high-risk features such as microcalcifications or irregular margins.

This study has several limitations. Firstly, as a retrospective surgical cohort, our study is subject to inherent selection bias. The observed malignancy rate of 48.8% is higher than the widely cited range of 10–40% for indeterminate thyroid nodules [29]. This reflects the real-world clinical practice where patients with suspicious ultrasound features or higher clinical suspicion are preferentially referred for surgery, while those with benign-appearing features are more likely to undergo active surveillance. Therefore, our findings are most applicable to patients with Bethesda III/IV nodules who are candidates for surgical intervention, rather than the entire population of patients with indeterminate cytology. Future work should involve larger-scale, multi-center prospective cohort studies for validation. The interpretation of ultrasound image features (particularly CEUS) and FNAB cytology involves a degree of subjectivity. Although all readings in this study were performed by senior physicians, inter-observer variability is inevitable. Secondly, the diagnosis of Bethesda III cytology itself carries inherent subjectivity, as it represents a heterogeneous category of “atypia of undetermined significance.” This subjectivity may have influenced patient selection for surgery and contributed to selection bias in our surgical cohort. It is recommended that subsequent research explore more quantitative analysis parameters (e.g., CEUS time–intensity curves) or employ AI algorithms to deeply mine radiomic features, thereby developing more objective and standardized diagnostic models. Thirdly, our multivariate logistic model was developed and tested within the same cohort without external validation. Although internal validation techniques such as bootstrapping or cross-validation can provide optimism-corrected performance estimates, these were not performed in the current study due to the relatively modest sample size and the exploratory nature of our analysis. Therefore, our findings should be interpreted with caution and considered hypothesis-generating rather than definitive. Finally, based on the above results, it is evident that conventional ultrasound remains the most fundamental and crucial component in evaluating such indeterminate thyroid nodules. In clinical practice, the advantages of physician experience and conventional ultrasound models should be fully leveraged for integrated interpretation. Future studies also need to explore more optimal feature fusion models to clarify the auxiliary diagnostic value of CEUS in specific thyroid nodule subgroups.

Furthermore, emerging evidence suggests that ultrasound radiomics—which enables high-throughput extraction of quantitative imaging features beyond human visual perception—holds promise for improving the diagnostic accuracy of thyroid nodules [30,31]. Future studies incorporating radiomics-based approaches may further refine risk stratification for cytologically indeterminate thyroid nodules by capturing subtle texture patterns that could complement conventional ultrasound and CEUS findings. For instance, a recent study by Chen et al. [30] demonstrated that ultrasound radiomics models could achieve a sensitivity of 90.5%, a positive predictive value of 75.0%, and reduce unnecessary biopsies by 21.1% in indeterminate thyroid nodules, highlighting the potential of objective quantitative metrics to enhance clinical decision-making. Zhou et al. [32] developed a deep learning radiomics model that achieved an AUC of 0.97 in external validation for differentiating benign from malignant thyroid nodules.

## 5. Conclusions

Patient age is an independent protective factor, while microcalcifications, irregular shape, and a taller-than-wide shape on conventional ultrasound are independent predictors of malignancy in Bethesda III/IV thyroid nodules. The addition of contrast-enhanced ultrasound does not significantly improve diagnostic performance beyond conventional ultrasound alone. Therefore, risk stratification based on these four independent factors remains the cornerstone for guiding clinical decision-making in patients with cytologically indeterminate thyroid nodules.

## Figures and Tables

**Table 1:** Baseline Characteristics and Univariate Analysis of Benign and Malignant Nodules.

Variable *n* (%)	Pathological Result	Statistic	χ^2^/t	*p*-Value
	Benign (*n* = 84)	Malignant (*n* = 80)		
Baseline Characteristics				
Age (years)	51.7 ± 9.3	44.5 ± 10.8	−4.538	<0.001 *
Gender			6.363	0.012 *
Male	18 (21.4)	6 (7.5)		
Female	66 (78.6)	74 (92.5)		
Maximum Diameter (cm)	13.3 ± 10.8	8.4 ± 7.2	−3.408	<0.001 *
TSH (mIU/L)	2.78 ± 2.24	2.67 ± 1.82	−0.331	0.741
Tg (μg/L)	440.3 ± 3137.9	52.7 ± 103.4	−0.995	0.323
Conventional Ultrasound Features				
Composition			3.905	0.048 *
Mixed solid-cystic	4 (4.8)	0 (0)		
Solid	80 (95.2)	80 (100)		
Echogenicity			6.303	0.012 *
Hypoechoic	73 (86.9)	78 (97.5)		
Other (Iso-/Hyperechoic)	11 (13.1)	2 (2.5)		
Echotexture Homogeneity			0.130	0.718
Heterogeneous	73 (86.9)	71 (88.8)		
Homogeneous	11 (13.1)	9 (11.3)		
Aspect Ratio			7.81	0.005 *
>1	28 (33.3)	44 (55.0)		
<1	56 (66.7)	36 (45.0)		
Margin			4.709	0.030 *
Ill-defined	32 (38.1)	44 (55.0)		
Well-defined	52 (61.9)	36 (45.0)		
Calcification Type			12.869	0.002 *
Microcalcifications	16 (19.0)	36 (45.0)		
Macrocalcifications	15 (17.9)	11 (13.8)		
No calcification	53 (63.1)	33 (41.3)		
Presence of Calcification			7.840	0.005 *
Yes	31 (36.9)	47 (58.8)		
No	53 (63.1)	33 (41.3)		
Shape			13.615	<0.001 *
Irregular	43 (51.2)	63 (78.8)		
Regular	41 (48.8)	17 (21.3)		
Halo Sign			6.964	0.008 *
Present	7 (8.3)	0 (0)		
Absent	77 (91.7)	80 (100)		
Focality			0.161	0.689
Single	33 (39.3)	29 (36.3)		
Multiple	51 (60.7)	51 (63.8)		
Hashimoto’s Thyroiditis			1.774	0.183
Yes	29 (34.5)	20 (25.0)		
No	55 (65.5)	60 (75.0)		
Location			1.615	0.446
Left Lobe	36 (42.9)	42 (52.5)		
Right Lobe	45 (53.6)	35 (43.8)		
Isthmus	3 (3.6)	3 (3.8)		
Contrast-Enhanced Ultrasound Features				
Enhancement Direction			4.595	0.100
Centripetal	29 (34.5)	37 (46.3)		
Centrifugal	21 (25.0)	23 (28.8)		
Diffuse	34 (40.5)	20 (25.0)		
Wash-in Speed			2.541	0.281
Fast	13 (15.5)	11 (13.8)		
Slow	46 (54.8)	53 (66.3)		
Synchronous	25 (29.8)	16 (20.0)		
Wash-out Speed			0.300	0.861
Fast	45 (53.6)	45 (56.3)		
Slow	7 (8.3)	5 (6.3)		
Synchronous	32 (38.1)	30 (37.5)		
Enhancement Homogeneity			0.513	0.474
Heterogeneous	61 (72.6)	54 (67.5)		
Homogeneous	23 (27.4)	26 (32.5)		
Enhancement Intensity			2.855	0.240
Hyper-enhancement	11 (13.1)	5 (6.3)		
Iso-enhancement	25 (29.8)	21 (26.3)		
Hypo-enhancement	48 (57.1)	54 (67.5)		
Rim Enhancement			3.733	0.053
Present	10 (11.9)	3 (3.8)		
Absent	74 (88.1)	77 (96.3)		
Enhancement Margin			9.232	0.002 *
Ill-defined	50 (59.5)	65 (81.3)		
Well-defined	34 (40.5)	15 (18.8)		

* *p <* 0.05 was considered statistically significant.

**Table 2:** Pathological Classification.

	Bethesda III (%)	Bethesda IV (%)	Total (%)
Benign Lesions			
Nodular Goiter	38 (26.2)	6 (31.6)	44 (26.8)
Hashimoto’s Thyroiditis	21 (14.5)	0 (0)	21 (12.8)
Follicular Adenoma	9 (6.2)	5 (26.3)	14 (8.5)
Subacute Thyroiditis	5 (3.4)	0 (0)	5 (3.0)
Benign Subtotal	73 (50.3)	11 (57.9)	84 (51.2)
Malignant Lesions			
Papillary Thyroid Carcinoma	69 (47.6)	8 (42.1)	77 (47.0)
Follicular Carcinoma	1 (0.7)	0 (0)	1 (0.6)
Medullary Thyroid Carcinoma	1 (0.7)	0 (0)	1 (0.6)
Lymphoma	1 (0.7)	0 (0)	1 (0.6)
Malignant Subtotal	72 (49.7)	8 (42.1)	80 (48.8)
Total	145 (100)	19 (100)	164 (100)

**Table 3:** Multivariate Analysis.

Variable	B	SE	Wald	df	*p*	OR	95%CI
III-defined enhancement margin	0.562	0.463	1.474	1	0.225	1.754	0.708–4.345
Gender	−0.724	0.576	1.580	1	0.209	0.485	0.157–1.499
Age	−0.077	0.021	13.523	1	<0.001	0.926	0.888–0.965
Maximum diameter	−0.038	0.034	1.282	1	0.258	0.963	0.901–1.028
Aspect ratio >1	0.916	0.418	4.800	1	0.028	2.499	1.101–5.672
Ill-defined margin	0.188	0.392	0.230	1	0.631	1.207	0.560–2.602
Calcifications			11.750	2	0.003		
Macrocalcifications	0.832	0.553	2.261	1	0.133	2.299	0.777–6.800
Microcalcifications	1.572	0.460	11.649	1	<0.001	4.815	1.953–11.871
Irregular shape	0.902	0.427	4.458	1	0.035	2.465	1.067–5.694
Constant	2.730	1.340	4.151	1	0.042	15.332	

Note: The reported mean, standard deviation, and corresponding *p*-value are for reference only; its distribution is better described using the median. The variables “halo” and “composition” demonstrated complete separation in the dataset (where all cases within a specific category belonged to the same pathological outcome). To avoid bias in model estimation, these variables were not included in the multivariate logistic regression analysis.

**Table 4:** Comparison of Diagnostic Performance of Different Models for Bethesda III/IV Thyroid Nodules.

Model	AUC (95% CI)	Optimal Cut-Off Point	Sensitivity (%)	Specificity (%)	PPV (%)	NPV (%)	Accuracy (%)
CUS Model	0.815 (0.752–0.879)	0.367	91.3	59.2	68.2	87.7	75.0
CEUS Model	0.609 (0.522–0.695)	0.436	81.3	40.5	56.5	69.4	60.4
Combined Model	0.823 (0.761–0.885)	0.411	83.8	61.9	67.7	80.0	72.6
Physician Diagnosis Model	0.775(0.701–0.849)	0.531	80.0	75.0	75.3	79.7	77.4

## Data Availability

Dataset available on request from the authors.

## Abbreviations

The following abbreviations are used in this manuscript:

CUS	Conventional Ultrasound
CEUS	Contrast-Enhanced Ultrasound
TNs	Thyroid Nodules
TI-RADS	Thyroid Imaging Reporting and Data System
FNAB	Fine-Needle Aspiration Biopsy
TBSRTC	The Bethesda System for Reporting Thyroid Cytopathology
ROC	Receiver Operating Characteristic
AUC	Area Under the Curve
PACS	Picture Archiving and Communication System
HIS	Hospital Information System
C-TIRADS	Chinese Thyroid Imaging Reporting and Data System
TSH	Thyroid-Stimulating Hormone
Tg	Thyroglobulin
OR	Odds Ratio
CI	Confidence Interval
NPV	Negative Predictive Value
PPV	Positive Predictive Value
AI	Artificial Intelligence
PTC	Papillary Thyroid Carcinoma
